# Therapeutic Effects of Hydrogen Gas Inhalation on Trimethyltin-Induced Neurotoxicity and Cognitive Impairment in the C57BL/6 Mice Model

**DOI:** 10.3390/ijms222413313

**Published:** 2021-12-10

**Authors:** Eun-Sook Jeong, Johny Bajgai, In-Soo You, Md. Habibur Rahman, Ailyn Fadriquela, Subham Sharma, Hwang-Un Kwon, So-Yeon Lee, Cheol-Su Kim, Kyu-Jae Lee

**Affiliations:** 1 Department of Environmental Medical Biology, Wonju College of Medicine, Yonsei University, Wonju 26426, Korea; micca08@naver.com (E.-S.J.); johnybajgai@yonsei.ac.kr (J.B.); pharmacisthabib@yonsei.ac.kr (M.H.R.); subhamsharma047@gmail.com (S.S.); cs-kim@yonsei.ac.kr (C.-S.K.); 2 GOOTZ Co., Ltd., 79-6, Yuljeong-ro 247 beon-gil, Yangju-si, Suwon 11457, Korea; igootz@naver.com (I.-S.Y.); kwon@mygootz.com (H.-U.K.); sylee@mygootz.com (S.-Y.L.); 3 Department of Global Medical Science, Wonju College of Medicine, Yonsei University, Wonju 26426, Korea; 4 Department of Laboratory Medicine, Wonju College of Medicine, Yonsei University, Wonju 26426, Korea; ailyn@yonsei.ac.kr

**Keywords:** molecular hydrogen, trimethyltin, oxidative stress, Alzheimer’s disease, cognitive dysfunction

## Abstract

Oxidative stress (OS) is one of the causative factors in the pathogenesis of various neurodegenerative diseases, including Alzheimer’s disease (AD) and cognitive dysfunction. In the present study, we investigated the effects of hydrogen (H_2_) gas inhalation in trimethyltin (TMT)-induced neurotoxicity and cognitive dysfunction in the C57BL/6 mice. First, mice were divided into the following groups: mice without TMT injection (NC), TMT-only injection group (TMT only), TMT injection + lithium chloride-treated group as a positive control (PC), and TMT injection + 2% H_2_ inhalation-treated group (H_2_). The TMT injection groups were administered a single dosage of intraperitoneal TMT injection (2.6 mg/kg body weight) and the H_2_ group was treated with 2% H_2_ for 30 min once a day for four weeks. Additionally, a behavioral test was performed with Y-maze to test the cognitive abilities of the mice. Furthermore, multiple OS- and AD-related biomarkers such as reactive oxygen species (ROS), nitric oxide (NO), calcium (Ca^2+^), malondialdehyde (MDA), glutathione peroxidase (GPx), catalase, inflammatory cytokines, apolipoprotein E (Apo-E), amyloid β (Aβ)-40, phospho-tau (p-tau), Bcl-2, and Bcl-2- associated X (Bax) were investigated in the blood and brain. Our results demonstrated that TMT exposure alters seizure and spatial recognition memory. However, after H_2_ treatment, memory deficits were ameliorated. H_2_ treatment also decreased AD-related biomarkers, such as Apo-E, Aβ-40, p-tau, and Bax and OS markers such as ROS, NO, Ca^2+^, and MDA in both serum and brain. In contrast, catalase and GPx activities were significantly increased in the TMT-only group and decreased after H_2_ gas treatment in serum and brain. In addition, inflammatory cytokines such as granulocyte colony-stimulating factors (G-CSF), interleukin (IL)-6, and tumor necrosis factor alpha (TNF-α) were found to be significantly decreased after H_2_ treatment in both serum and brain lysates. In contrast, Bcl-2 and vascular endothelial growth factor (VEGF) expression levels were found to be enhanced after H_2_ treatment. Taken together, our results demonstrated that 2% H_2_ gas inhalation in TMT-treated mice exhibits memory enhancing activity and decreases the AD, OS, and inflammatory-related markers. Therefore, H_2_ might be a candidate for repairing neurodegenerative diseases with cognitive dysfunction. However, further mechanistic studies are needed to fully clarify the effects of H_2_ inhalation on TMT-induced neurotoxicity and cognitive dysfunction.

## 1. Introduction

Neurodegenerative diseases are groups of different aging disorders such as Alzheimer’s disease (AD), Parkinson’s disease, amyotrophic lateral sclerosis, and Huntington’s disease, which gradually lead to an increase in neuronal cells, the accumulation of abnormally aggregated proteins, and apoptosis, ultimately affecting the motor and cognitive function of an individual [1]. AD is the most extensively reported neurodegenerative disease to date and is associated with complex and multifactorial pathophysiology [2,3]. Although there are several factors associated with the development of AD, amyloid β (Aβ) and tau protein are most closely associated with its pathogenesis [4,5,6]. Aβ abnormally accumulates in AD brain tissues and forms extracellular plaques that are known to induce synaptic alterations and neurodegeneration and may contribute to cognitive deficit [4]. On the other hand, hyperphosphorylation of tau protein forms intracellular neurofibrillary tangles (NFT) and is strongly responsible for neurodegeneration [5,6]. In addition, increasing evidence shows that oxidative stress (OS) plays a significant role in the progression of neurodegenerative diseases and neurodegeneration, as it contributes to the destruction of membranes, cytoskeleton alterations, and apoptosis due to excessive production of reactive oxygen species (ROS), reactive nitrogen species (RNS), and inflammatory mediators such as inflammatory cytokines [1,7,8,9,10]. In AD, due to oxidative imbalance-mediated injury to the brain region, lipid peroxidation markers are found to be elevated and extensively studied in its pathogenesis [9]. Therefore, OS may easily cause degeneration in the hippocampal region in brain, which is the major region for memory processing [11,12]. Thus, to ameliorate neurodegenerative diseases such as AD, different therapeutic approaches are developing at a rapid pace. Therefore, several animal models, such as TMT-induced neurodegeneration are often used for exploring the effects of therapeutic candidates on neurodegenerative diseases such as AD.

Trimethyltin (TMT) is a neurotoxin and organometallic compound that induces substantial neurodegeneration and neuronal cell death in the central nervous system (CNS), particularly in the hippocampus region in the brain [13]. Several studies have shown that the systemic administration of TMT in human and mice demonstrated clinical symptoms, such as hyperactivity, aggressiveness, cognitive deficits, and seizure-like behaviors [13,14,15]. However, evidence shows that all of these symptoms are associated with AD [2,6]. Additionally, previously conducted experimental observations suggest that TMT-induction induces neuronal cell death, intracellular calcium (Ca^2+^) overload, mitochondrial damage, and OS [16,17,18]. Moreover, TMT-induction enhances neurogenesis in the brain and increases the expression of the amyloid precursor protein, several signaling pathways, inflammatory cytokines such as tumor necrosis factor alpha (TNF-α), interleukin (IL)-1β, and nitric oxide (NO) levels in the hippocampus region, and might be involved in brain injury [19,20,21,22]. Due to this reason, the TMT-induced animal model is widely used in research for investigating brain dysfunction and neurodegeneration [11,23]. From these background studies, it is important to find an excellent multi-potent agent with antioxidant and anti-inflammatory properties for repairing the brain cells against TMT-induced neurotoxicity and cognitive impairment.

Molecular hydrogen (H_2_) is potential novel antioxidant, and previously published studies have strongly suggested its potential for preventive and therapeutic applications [24,25,26]. In recent years, research on H_2_ therapy for neurodegenerative diseases such as AD has become a hotspot due to its anti-oxidative, anti-apoptotic, and anti-inflammatory effects [25,27]. So far, several animal experiments and more than 25 double-blinded clinical studies have been reported regarding the efficacies of H_2_ [25,26]. Administration of H_2_ gas by inhalation can readily penetrate through biomembranes, such as the blood–brain barrier (BBB) and the placental barrier owing to its small molecular size, non-ionic state, and hydrophobic properties, thus benefiting complex organs such as the brain and organelles [28,29]. Several studies have reported that H_2_ plays a positive role in neuroprotection under conditions such as cerebral infarction, Parkinson’s disease, cognitive impairment, and brain injury by attenuating OS and the inflammatory response [26,28,30,31,32,33,34]. In previous studies conducted using an inflammatory disease model, the administration of H_2_ caused anti-inflammatory response by ameliorating the pro-inflammatory markers, such as TNF-α, IL-1β, and IL-6 [26,30,32]. In addition, Iuchi and colleagues demonstrated that administration of H_2_ (approximately 1%, *v*/*v*) modulated the Ca^2+^ signal and modified the generation of oxidized phospholipid species [35]. Recently, Zhou and colleagues reported that hydrogen-rich water has potential to ameliorate TMT-induced memory impairment by regulating Siah E3 ubiquitin protein ligase 1 [36]. However, the therapeutic effects of H_2_ on chemically induced neurodegenerative diseases model such as the TMT-induced animal model are poorly studied. Consequently, the actual molecular mechanism through which H_2_ treatment induces CNS protection against TMT-induced neurotoxicity and cognitive dysfunction has not been yet fully elucidated. Therefore, based on the neurodegenerative and oxidative damage of TMT, as well as the antioxidative and anti-inflammatory characteristics of hydrogen, the present study aimed to investigate the potential therapeutic effects of 2% H_2_ gas inhalation on TMT-induced neurotoxicity and cognitive impairment in a TMT-induced neurodegenerative mouse model. In addition, we explored the effects of H_2_ in the TMT-induced oxidative and inflammatory response in serum and brain lysates.

## 2. Results

### 2.1. Effects of H_2_ Gas Inhalation in TMT-Induced C57BL/6 Mice on Seizure Behavior and Body Weight Measurement

**Figure 1:**
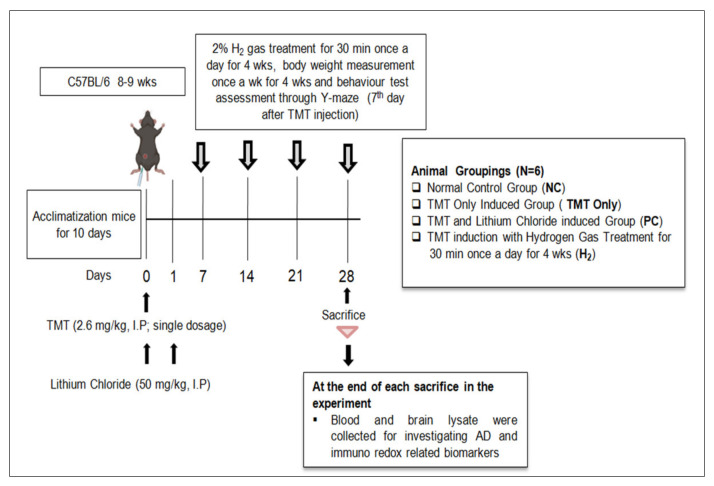
Schematic diagram of the experimental process.

**Figure 2:**
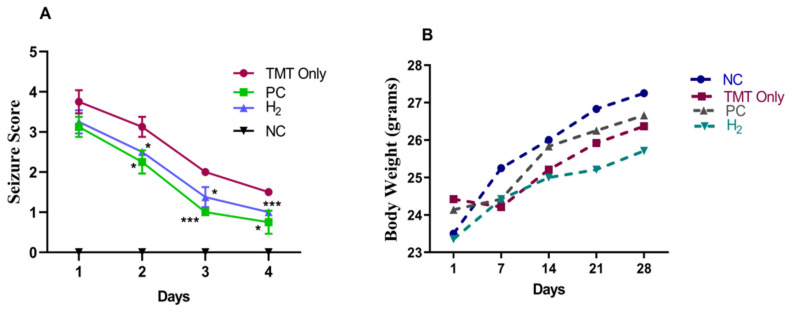
Effects of H_2_ gas inhalation treatment on the seizure behavior (**A**) and body weight (**B**) of the TMT-induced C57BL/6 mice. Data are shown as the mean ± SEM for n = 4 mice. Significant differences were analyzed with ANOVA and Tukey’s test. * *p* < 0.05, *** *p* < 0.001 compared with TMT-only group.

The TMT injection, H_2_ administration, and all experimental procedures were conducted following the timeline indicated in Figure 1. TMT induction is known to induce seizure behavior in mice [13]. In our study, we tested the effects of H_2_ gas inhalation on seizure-like behaviors in TMT-induced mice. During the experiment, with TMT induction, symptoms such as tremors, seizures, and aggressive behavior were observed in mice for four days. However, administration of PC and H_2_ treatment resulted in a significant decrease in seizure-like behaviors from day 2 to 4 (Figure 2A). On day 2 (*p* < 0.05), day 3 (*p* < 0.001), and day 4 (*p* < 0.01), the behavioral seizure score showed a significant reduction in the PC-treated group compared to the TMT-only group. Likewise, administration of H_2_ also significantly decreased seizure-like behaviors in TMT-induced mice from day 2 (*p* < 0.05), day 3 (*p* < 0.05), and day 4 (*p* < 0.001) compared to the TMT-only group (Figure 2A). These results indicate that administration of H_2_ gas inhalation reduced seizure-like behaviors in TMT-induced mice. Additionally, the body weight was measured once per week for four weeks to obtain baseline data. Our results showed that TMT induction led to a slight decrease in body weight on the 7th day in the TMT-only group compared to the NC, PC, and H_2_ groups. However, the difference was not significant (Figure 2B).

### 2.2. Effects of H_2_ Gas Inhalation on TMT-Induced Cognitive Dysfunction

**Figure 3:**
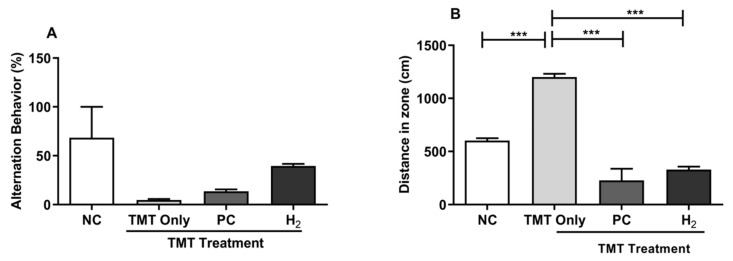
Effects of H_2_ gas inhalation treatment on TMT-induced spatial cognitive impairment in C57BL/6 mice. After TMT induction (2.6 mg/kg body weight), 2% H_2_ gas and lithium chloride (PC, 50 mg/kg body weight) were ip administered to six mice. (**A**) Alternation behavior on day 7. (**B**) Distance traveled in the Y-maze compartment. Data are shown as the mean ± SEM for n = 4 mice. Significant differences were analyzed with ANOVA and Tukey’s test. *** *p* < 0.001.

To investigate the effects of H_2_ gas inhalation on TMT-induced cognitive dysfunction, TMT was injected intraperitoneally (ip) in mice, and 2% H_2_ gas was administered for four weeks. Since the Y-maze test was conducted to determine spatial cognition and short-term abilities, we performed the Y-maze test on day 7 after TMT induction to determine the willingness of mice to explore new circumstances and to assess their short-term memory. We found that spontaneous alternation (%) of the TMT-only group was decreased compared to the NC-, PC-, and H_2_-treated groups (Figure 3A). In addition, we found significantly increased distance travel in the maze compartment zone in the TMT-only group (*p* < 0.001) compared to that in the NC-, PC-, and H_2_-treated groups (Figure 3B). A previous study reported that TMT-exposed animals showed cognitive impairment as well as hyperactive behavior compared to the control group [13], which is consistent with our findings in this study. Therefore, our results suggest that H_2_ can attenuate the TMT-induced impairment of the spatial working memory and hyperactivity in mice. However, more types of maze experiments are needed to fully confirm this notion.

### 2.3. Effects of H_2_ Gas Inhalation on TMT-Induced ROS and NO Levels in Mice Serum and Brain

**Figure 4:**
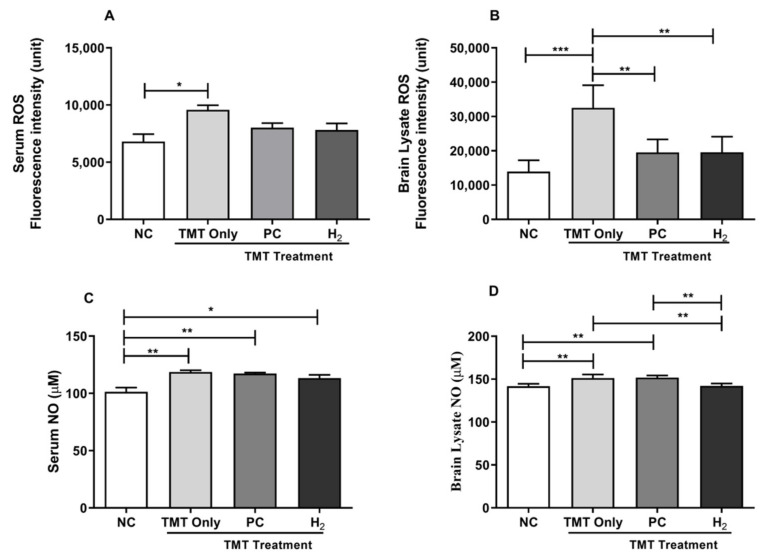
Effects of H_2_ gas inhalation treatment on total ROS and NO levels in TMT-induced C57BL/6 mice after four weeks. (**A**) Serum ROS, (**B**) Brain lysates ROS, (**C**) Serum NO, and (**D**) Brain lysates NO levels. Data are shown as the mean ± SEM for n = 4 mice. Significant differences were analyzed with ANOVA and Tukey’s test. * *p* < 0.05, ** *p* < 0.01, *** *p* < 0.001.

OS is considered one of the known toxic mechanisms of TMT induction [13]. Thus, to investigate the effects of H_2_ gas inhalation on TMT-induced neuronal toxicity, we analyzed the total ROS and NO levels in both the serum and brain lysates of C57BL/6 mice. Our results showed a significant increase in total intracellular ROS levels (*p* < 0.05) and NO levels (*p* < 0.01) in the serum of the TMT-only group compared to the NC group (Figure 4A,C). Additionally, we found that the ROS and NO levels were markedly increased in the brain lysates of the TMT-only group (*p* < 0.001, and *p* < 0.01) compared with the NC group (Figure 4B,D). In contrast, ROS levels were significantly decreased in the brain of the H_2_-treated (*p* < 0.01) and PC-treated groups (*p* < 0.01) compared with the TMT-only group (Figure 4B). Additionally, upon H_2_ treatment, TMT-induced NO levels were significantly decreased (*p* < 0.01) compared to the TMT-only group (Figure 4D). Taken together, our results showed that with H_2_ gas treatment, both OS markers, i.e., ROS and NO, were effective in repairing TMT-induced neuronal damage.

### 2.4. Effects of H_2_ Gas Inhalation on TMT-Induced Ca^2+^ and Malondialdehyde Levels in Mice Serum and Brain

**Figure 5:**
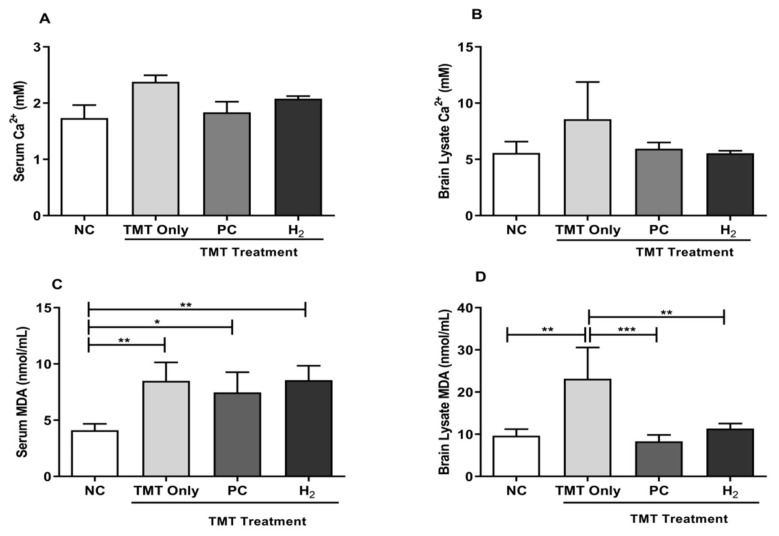
Effects of H_2_ gas inhalation treatment on Ca^2+^ and MDA levels in TMT-induced C57BL/6 mice after four weeks. (**A**) Serum Ca^2+^ level, (**B**) Brain lysate Ca^2+^ level, (**C**) Serum MDA level, (**D**) Brain lysate MDA level. Data are shown as the mean ± SEM for n = 4 mice. Significant differences were analyzed with ANOVA and Tukey’s test. * *p* < 0.05, ** *p* < 0.01, *** *p* < 0.001.

The role of lipid peroxidation and intracellular Ca^2+^ has been extensively studied in AD pathogenesis [11,16]. Malondialdehyde (MDA) is an indicator of lipid peroxidation induced by OS [9]. In our study, we investigated the effects of H_2_ inhalation on TMT-induced Ca^2+^ and lipid peroxidation levels in C57BL/6 mice in both the serum and brain lysates. As shown in Figure 5A,B, the Ca^2+^ level in both the serum and brain lysates of the TMT-only mice showed higher values compared to PC- and H_2_-treated mice. However, with PC and H_2_ treatment, the Ca^2+^ level was found to be decreased in both groups compared to the TMT-only group but without significant differences in both the serum and brain lysates. Furthermore, we evaluated the effects of H_2_ inhalation on the TMT-induced MDA level. As shown in Figure 5C, the MDA level in the TMT-only (*p* < 0.01), PC (*p* < 0.05), and H_2_ (*p* < 0.01) groups was significantly increased in the serum of TMT-injected mice compared with those of the NC mice. In contrast, we found a significantly increased MDA level in the TMT-only group in brain lysates compared with those of the NC mice. However, with PC and H_2_ administration, the level of MDA was significantly decreased in the PC- (*p* < 0.01) and H_2_-treated (*p* < 0.001) groups compared with the TMT-only group (Figure 5D). These results demonstrated that TMT effectively induced damage in the brain by increasing intracellular Ca^2+^ and MDA levels, while H_2_ treatment effectively repaired the damage caused by TMT and might have therapeutic effects.

### 2.5. Effects of H_2_ Gas Inhalation on Antioxidative Enzyme Activities in Mice Serum and Brain under TMT-Induced Damage 

**Figure 6:**
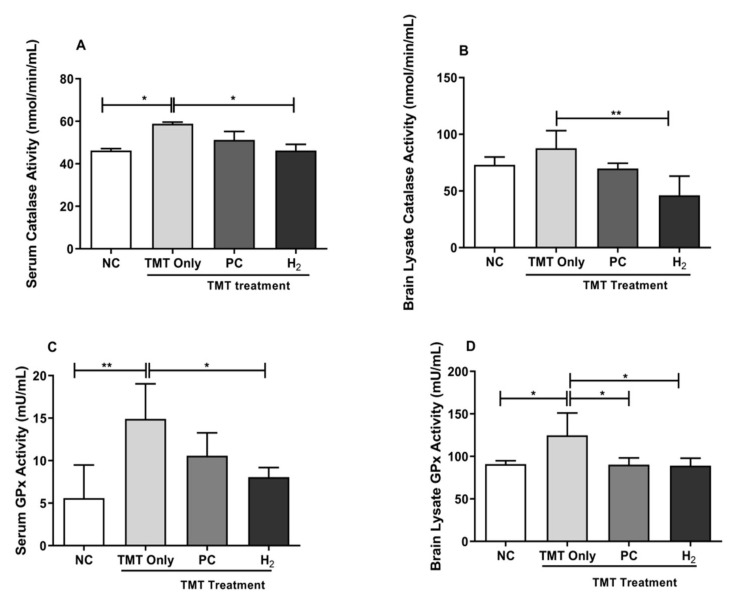
Effects of H_2_ gas inhalation treatment on total intracellular catalase and GPx levels in TMT-induced C57BL/6 mice after four weeks. (**A**) Serum catalase level, (**B**) Brain lysate catalase level, (**C**) Serum GPx level, (**D**) Brain lysate GPx level. Data are shown as the mean ± SEM for n = 4 mice. Significant differences were analyzed with ANOVA and Tukey’s test. * *p* < 0.05, ** *p* < 0.01.

To investigate the effects of H_2_ gas inhalation on OS imbalance under TMT-induced damage, we measured the activities of antioxidative enzymes, such as catalase and glutathione peroxidase activity (GPx), in the serum and brain lysates. Our results showed that catalase activity in serum was significantly decreased in the NC- (*p* < 0.05) and H_2_-treated groups (*p* < 0.05) compared with those of the TMT-only group (Figure 6A). In addition, our brain lysate result showed a significantly decreased catalase level in H_2_-treated group (*p* < 0.01) compared with the TMT-only group (Figure 6B). Moreover, we investigated the GPx level in both the serum and brain lysates. Our results showed significantly decreased GPx activity in both the NC- (*p* < 0.01) and H_2_-treated (*p* < 0.05) groups in serum (Figure 6C). Likewise, we found significantly decreased GPx activity in the brain lysates of the NC- (*p* < 0.05), PC- (*p* < 0.05), and H_2_-treated (*p* < 0.05) groups compared with the TMT-only group (Figure 6D). These results demonstrated that elevated catalase and GPx levels in the serum and brain with TMT induction may reflect a compensatory rise in antioxidants in response to oxidative damage.

### 2.6. Effects of H_2_ Gas Inhalation on Inflammatory Cytokines and Vascular Endothelial Growth Factor in Mice Serum and Brain under TMT-Induced Damage 

**Figure 7:**
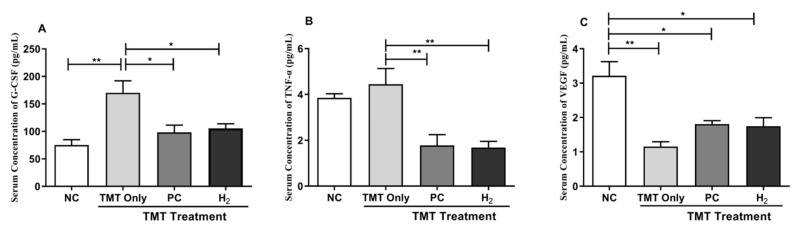
Effects of H_2_ gas inhalation treatment on inflammatory cytokines and VEGF in serum in TMT-induced C57BL/6 mice after four weeks. (**A**) G-CSF, (**B**) TNF-α, (**C**) VEGF. Data are shown as the mean ± SEM for n = 4 mice. Significant differences were analyzed with ANOVA and Tukey’s test. * *p* < 0.05, ** *p* < 0.01.

It is well established that TMT-induction plays a role in neuroinflammation [13,23]. Therefore, we investigated the expression of inflammatory cytokines, such as granulocyte colony-stimulating factors (G-CSF) and TNF-α, and vascular endothelial growth factor (VEGF) in the serum and brain lysates. Our results showed that the administration of TMT significantly decreased the G-CSF level in serum compared with the NC- (*p* < 0.01), PC- (*p* < 0.05), and H_2_-treated (*p* < 0.05) groups (Figure 7A). In addition, TNF-α level was significantly increased in the TMT-only group; however, with PC and H_2_ treatment, the level of TNF-α (inflammatory cytokines) in serum was significantly decreased in the PC- (*p* < 0.01) and H_2_-treated (*p* < 0.01) groups (Figure 7B). Moreover, we measured the VEGF level in the serum of mice. Our results showed that the level of VEGF was significantly decreased in the TMT-only group (*p* < 0.01) and the PC- (*p* < 0.05) and H_2_-treated (*p* < 0.05) groups compared with the NC group (Figure 7C).

**Figure 8:**
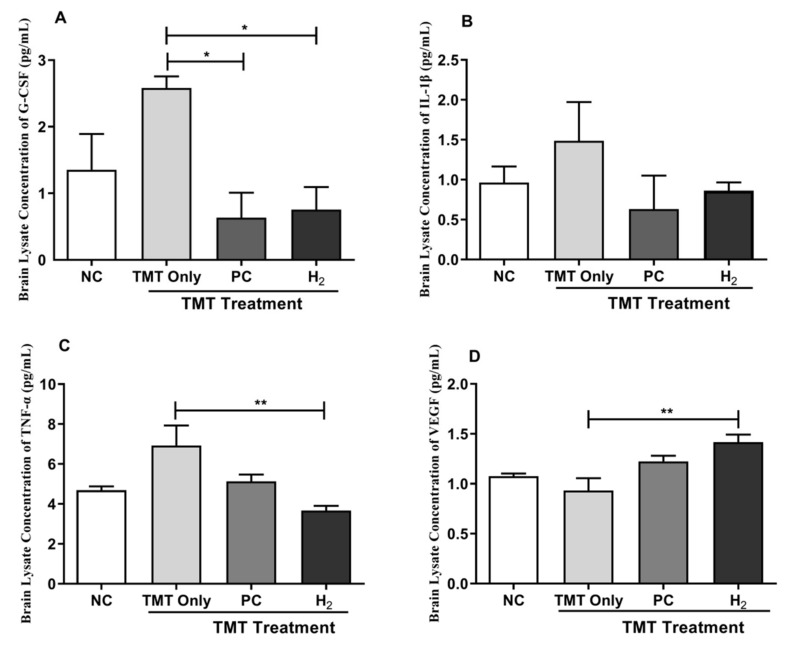
Effects of H_2_ gas inhalation treatment on inflammatory cytokines and VEGF level in brain lysates in TMT-induced C57BL/6 mice after four weeks. (**A**) G-CSF, (**B**) IL-1β, (**C**) TNF-α, (**D**) VEGF. Data are shown as the mean ± SEM for n = 4 mice. Significant differences were analyzed with ANOVA and Tukey’s test. * *p* < 0.05, ** *p* < 0.01.

Furthermore, our brain lysates results showed a significant reduction in the G-CSF level in the PC-treated (*p* < 0.05) and H_2_- treated (*p* < 0.05) groups compared with the TMT-only group (Figure 8A). In addition, the level of IL-1β cytokine was markedly increased in the TMT-only group but decreased in the NC, PC, and H_2_ -treated groups without a significant difference (Figure 8B). Moreover, the TNF-α level was significantly increased in the TMT-only group; however, with PC and H_2_ treatment, the level of TNF-α in the brain lysates was significantly decreased in the H_2_-treated group (*p* < 0.01) (Figure 8C). Additionally, VEGF expression was significantly increased in the H_2_-treated group compared to the TMT-only group (Figure 8D). Collectively, these results indicate that H_2_ effectively attenuated TMT-induced neurotoxicity and cognitive damage by inhibiting inflammatory cytokines and upregulating VEGF.

### 2.7. Effects of H_2_ Gas Inhalation on Intracellular Apolipoprotein E Activities in Mice Serum and Brain under TMT-Induced Damage 

**Figure 9:**
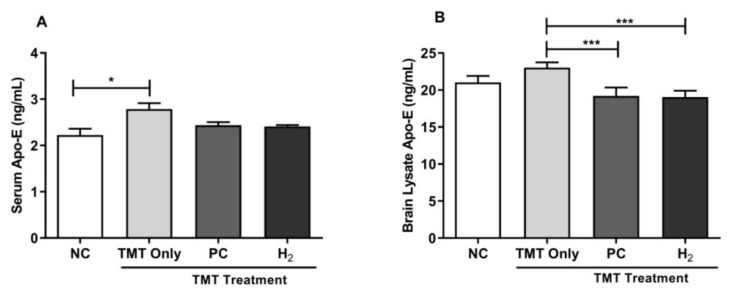
Effects of H_2_ gas inhalation treatment on intracellular Apo-E activities in TMT-induced C57BL/6 mice after four weeks. (**A**) Serum Apo-E level, (**B**) Brain lysate Apo-E level. Data are shown as the mean ± SEM for n = 4 mice. Significant differences were analyzed with ANOVA and Tukey’s test. * *p* < 0.05, *** *p* < 0.001.

The apolipoprotein E (Apo-E) gene is strongly associated with the risk of AD [37]. To verify the role of TMT-induction in Apo-E activity, we further evaluated total Apo-E levels in blood and brain lysates using ELISA. We found that Apo-E levels in serum were significantly increased in the TMT-only group (*p* < 0.05) compared to the NC group, whereas in the brain lysate, Apo-E activity was significantly decreased in the PC- (*p* < 0.001) and H_2_- treated (*p* < 0.001) groups compared with the TMT-only group (Figure 9A,B). Taken together, our results showed that TMT induction of Apo-E levels effectively induced brain damage and was associated with AD progression, whereas H_2_ treatment effectively improved brain damage associated with progression of TMT-induced AD.

### 2.8. Effects of H_2_ Gas Inhalation on AD-Related Biomarkers in Brain Tissue under TMT-Induced Damage

**Figure 10:**
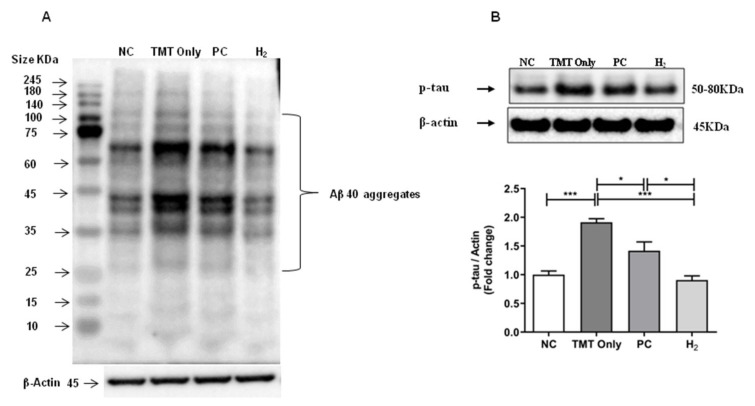
Effects of H_2_ gas inhalation treatment on the Aβ-40 (mouse monoclonal- 32A1) and p-tau-(Ser404) protein levels in brain lysates in TMT-induced C57BL/6 mice after four weeks. (**A**) The antibody-reactive bands of mouse Aβ-40 in the brain lysates, (**B**) The antibody-reactive band of p-tau-(Ser404) protein levels in brain lysates. The bar graphs represent the band intensity of each protein marker normalized to the total. The data are presented as the mean ± SD of n=3 mice. Significant differences were analyzed with ANOVA and Tukey’s test. * *p* < 0.05, *** *p* < 0.001.

We verified the underlying molecular mechanisms of H_2_ gas inhalation on TMT-induced neurotoxicity and cognitive impairment and evaluated the protein expression of AD-related biomarkers such as Aβ-40 and phospho-tau (p-tau)-(Ser404) by western blot analysis. In our results, we observed various sizes of bands of Aβ oligomers/aggregates in mouse brain lysates. In addition, we observed that a wide range of Aβ molecules such as oligomers and aggregates with increasing molecular size was intensified in the TMT-induced group compared to the NC group, indicating that AD was induced in the hippocampus following TMT induction. However, with PC and H_2_ treatment, the expressions of Aβ-40 oligomers/aggregates were found to be decreased compared to the TMT-only group (Figure 10A). Similarly, we found the expression levels of p-tau-(Ser404) were significantly increased in the TMT-only group (*p* < 0.001) compared with the NC group; however, with PC (*p* < 0.05) and H_2_ (*p* < 0.001) treatment, the expression of p-tau-(Ser404) was significantly decreased (Figure 10B).

### 2.9. Effects of H_2_ Gas Inhalation on Bcl-2 and Bcl-Associated X Protein in Brain Tissue under TMT-Induced Damage

**Figure 11:**
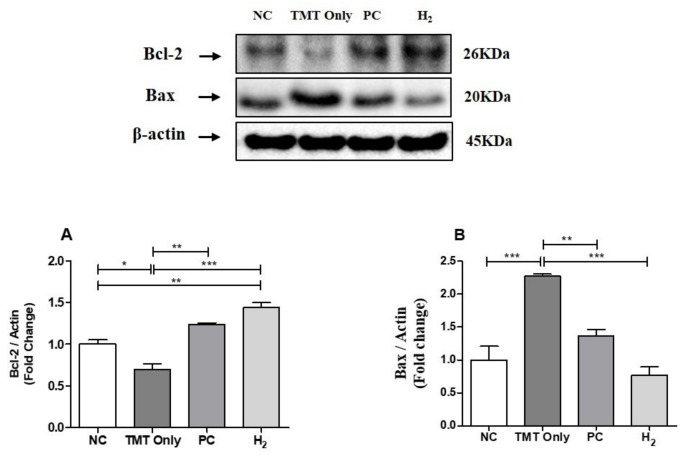
Effects of H_2_ gas inhalation treatment on the Bcl-2 and Bax protein levels in brain lysates of TMT-induced C57BL/6 mice after four weeks. (**A**) The antibody-reactive quantified band intensity of Bcl-2 in the brain lysates, (**B**) The antibody-reactive quantified band intensity of Bax protein in brain lysates. The bar graphs represent the band intensity of each protein marker normalized to the total. The data are presented as the mean ± SD for n = 3 mice. Significant differences were analyzed with ANOVA and Tukey’s test. * *p* < 0.05, ** *p* < 0.01, *** *p* < 0.001.

We investigated the underlying molecular mechanisms of H_2_ gas inhalation against TMT-induced neurotoxicity and evaluated the protein expression of Bcl-2- and Bcl-2- associated X (Bax) protein by western blot analysis. We found that the expression level of Bcl-2 was significantly decreased in the TMT-only group (*p* < 0.001) compared to the NC group. However, with PC (*p* < 0.01) and H_2_ (*p* < 0.001) treatment, the expression of Bcl-2 was significantly increased (Figure 11A). In contrast, we found that the expression levels of Bax were significantly increased in the TMT-only group (*p* < 0.001) compared to the NC group; however, with PC (*p* < 0.01) and H_2_ (*p* < 0.001) treatment, the expression of Bax was significantly decreased compared to the TMT-only group (Figure 11B), indicating that TMT-induced apoptosis was attenuated by H_2_ gas inhalation treatment in the brain.

## 3. Discussion

The present study investigated the therapeutic effects of H_2_ gas inhalation on TMT-induced neurotoxicity and cognitive impairment in mice. Our findings indicate that H_2_ gas treatment ameliorated TMT-induced OS and inflammatory and AD-related markers in C57BL/6 mice. Several studies have reported that neurodegenerative diseases are characterized by progressive neuronal death, resulting in the loss of neurons in specific areas, such as the hippocampal region of the brain, causing spatial cognitive impairment such as AD [2,3,38]. Recently, research in the H_2_ field has been emerging due to its high biosafety and effectiveness as a medical gas [39,40]. Studies have shown that H_2_ is highly capable of penetrating the biological membranes including the BBB. Thus, this characteristic of H_2_ provides unrestricted access to the CNS, causing therapeutic effects on many CNS diseases, including ischemia stroke, intracranial hemorrhage, and neurodegenerative diseases [41,42,43,44]. Moreover, previous studies have shown that administration of H_2_ inhalation attenuated redox stress, inflammatory and OS mediators, and prevented BBB disruption by reducing the activation of mast cells and degranulation that result in amelioration of neurological conditions [24,39,41]. In addition, studies have shown that inhalation of H_2_ gas (1–4%) was effective for the improvement of acute and chronic neurological conditions in different clinical and animal models [31,39]. Additionally, one of the recent studies has confirmed the safety and effectiveness of administration of 3% H_2_ on acute cerebral infarction patients [31]. Moreover, administration of 2% H_2_ gas inhalation alleviated BBB impairment and memory dysfunction in the brain through regulation of antioxidative pathways in mice [45]. Therefore, the attenuation of neuronal death through H_2_ treatment might be a novel strategy for the prevention and treatment of cognitive impairments such as AD.

TMT is a commonly used neurotoxin for constructing in vivo models of neurodegenerative diseases such as AD since TMT injection can specifically alter conditions such as tremors, seizures, and spatial recognition memory, which are associated with hippocampal dysfunction in the brain. Animals exposed to TMT experience spontaneous seizures and elevated neuronal excitability [13,15,16]. Interestingly, studies have reported that H_2_ has potential to protect against many neurological diseases, including cerebral injury, neuropathic pain, cognitive impairment, and AD [32,46,47,48]. Our results showed that TMT induction induces aggressive behavior, seizures, whole body tremors, and memory deficits in mice. Moreover, reduction in seizure behavior and improved memory were observed following repeated H_2_ gas inhalation in mice. Therefore, these results suggest that repeated administration of H_2_ gas attenuates TMT-induced toxicity in the hippocampal region of the brain. However, detailed investigations are required to verify this claim.

Compelling evidences suggest that extracellular Aβ plaques and hyperphosphorylated tau protein are the key pathological hallmarks in AD [4,5,6]. The deposition and aggregation of Aβ plaques and hyperphosphorylated tau proteins lead to excessive production of ROS and RNS that cause OS, chronic neuroinflammation, and dysfunction in mitochondrial function in the brain, causing neuronal loss and protein misfolding [49,50]. In TMT-induced neurotoxicity and damage, OS is considered one of the main mechanisms underlying neurodegeneration. Previous evidences have shown that TMT induces ROS, RNS, and MDA (lipid peroxidation marker) both in vivo and in vitro [51,52,53]. These oxidative conditions could affect behavioral abnormalities and imbalance of the antioxidant defense system and consequently cause neuronal cell death in TMT-induced animals [51,52]. In addition, endogenous compensatory antioxidative responses are activated simultaneously in the brain for the recovery of various neurological damage conditions, such as TMT induction. A previous study showed that H_2_ selectively reduced highly toxic ROS such as hydroxyl radical and peroxynitrite and protected the cells against OS [24]. In addition, another study showed that administration of H_2_ decreased OS-related markers such as hydroxyl-2-nonenal, NO, and MDA in patients and rodents [54]. In our study, we observed a significant increase in intracellular ROS, NO, and MDA levels in the TMT-induced mice, whereas H_2_ gas treatment significantly attenuated these oxidative markers in both the brain and blood. In contrast, our results showed that the activities of antioxidant enzymes such as catalase and GPx were increased in the TMT-induced group and decreased in the PC and H_2_ groups. This might be due to the compensatory mechanism of the antioxidant defense system against TMT-induced damage. Moreover, previous publications on hydrogen effects have reported that the administration of H_2_ alleviates neurotoxicity by decreasing levels of OS biomarkers [3,25]. Several studies have reported a connection between Ca^2+^ homeostasis disruption and the development of neuro-diseases such as AD [55,56]. Moreover, at the cellular level, it has been reported that abnormal amyloid accumulation induces an upregulation of neuronal Ca^2+^ signaling. Excessive Ca^2+^ release from the endoplasmic reticulum plays an important role in orchestrating the dynamics of the neuropathology of AD and associated memory loss and cognitive dysfunction [55,56]. Additionally, one study reported that TMT-intoxicated injection dysregulated intracellular Ca^2+^ in hippocampal neurons [15]. Toward this, a previous study conducted on hydrogen administration demonstrated that exposure of cultured cells to H_2_-dependently autoxidized phospholipid species reduced the Ca^2+^ signal and mediated the expression of different genes [35]. Our present results revealed that TMT-induction increased the values of intracellular Ca^2+^ activity in both the serum and brain tissue. In addition, our findings show that H_2_ gas administration reduced the values of the upregulated intercellular Ca^2+^ activity in the blood and brain. Taken together, these results indicate that administration of H_2_ could effectively attenuate the OS markers both systematically and induced by TMT intoxication in mice brain, as well as aid recovery from TMT-induced neuronal damage.

Increasing evidence shows that neuroinflammation plays a vital role in the progression of AD pathogenesis. Inflammation is not only known to be the key pathophysiological mechanism of Aβ plaques formation, but it is also a main cause of tau hyperphosphorylation, NFT formation, and neurodegeneration [57,58]. Chronically, neurotoxins such as Aβ can activate microglia to release cytotoxic factors, such as superoxide (O_2_^−^), NO, TNF-α, and IL-1β in the brain that reliably trigger neuronal death [59,60]. Another study reported that patients with AD had higher levels of pro-inflammatory cytokines, such as IL-4, IL-10, G-CSF, monocyte chemotactic protein 1, and TNF-α [61]. In contrast, VEGF signaling proteins have been reported to have protective effects against cognitive impairment [62]. Additionally, a study reported that TMT intoxication in mice induced neuronal death and the enhancement of inflammatory factors, including TNF-α and IL-1β, in the hippocampus [60]. Studies on H_2_ have reported anti-inflammatory actions [32,54,63]. A recently published study on H_2_ also demonstrated anti-inflammatory action in 3 × Tg-AD mice [64]. In the present study, we examined cytokines such as G-CSF, IL-1β, TNF-α, and VEGF in blood and brain tissues to investigate the effects of H_2_ administration on TMT-induced cognitive damage. Our findings indicate that TMT intoxication in mice significantly increased the levels of inflammatory cytokines such as G-CSF and TNF-α in the blood, which were attenuated following four weeks of H_2_ gas administration. Additionally, H_2_ treatment inhibited the expression of inflammatory cytokines, such as G-CSF, IL-1β, and TNF-α, in the brain tissue induced with TMT. Moreover, we found that H_2_ treatment increased the VEGF protein levels in both blood and brain tissues in mice under TMT-induction. Thus, we may conclude that H_2_ administration in TMT-induced mice exhibited repairing effects, which resulted in reduced TMT-induced neurotoxic damage by inhibiting inflammatory mediators, such as inflammatory cytokines, and improving cognitive function.

Furthermore, a previous study revealed that the Apo-E protein is associated with a greater memory decline rate and cognitive dysfunction in patients with mild cognitive impairment [65]. Apo-E plays an important role in tau pathology, in the presence of Aβ, in the progression of AD, and in the decline of cognitive function [66]. Additionally, it is known that accumulation and fibrillary deposition of the Aβ peptide in senile plaques and tau phosphorylation in neurofibrillary tangles are the major causes of cognitive dysfunction. In addition, toxic oligomers are generated in brain by accumulation of Aβ, which triggered OS-induced damage, neuroinflammation, hyperphosphorylation of tau, and dysregulation of metabolic pathways [4]. A study conducted by Park et al., reported that TMT induction increased the p-tau expression in TMT-induced mice [67]. Accordingly, a previous conducted a study on H_2_ showed amelioration of TMT-induced spatial learning and memory impairment in mice by regulation of Siah-1 [36]. In the present study, we evaluated the cognitive improvement effects of H_2_ in TMT-induced mice by measuring the Apo-E levels in the blood and brain. Our results revealed a significant increase in Apo-E levels in both the blood and brain of TMT-induced mice. On the other hand, H_2_ administration in mice for four weeks after TMT-induction significantly decreased Apo-E levels in the brain. Additionally, we measured Aβ-40 and p-tau protein expressions using western blot in the TMT-induced hippocampal brain samples. Our results showed that Aβ-40 and p-tau protein expressions were significantly increased in the TMT-only group, whereas with H_2_ and PC treatment, expressions of Aβ-40 and p-tau were significantly attenuated in TMT-induced mice.

It is well known that apoptosis is an important mechanism for TMT-induced hippocampal neuronal toxicity and damage [13,16,17]. Bcl-2 and Bax play important roles in promoting apoptosis whereas their roles are functionally opposite. Bcl-2 is known as the key cell apoptosis protein marker, whereas Bax acts to promote apoptosis. In a previous study, administration of hydrogen-rich saline showed improved neurological function and decreased neuronal apoptosis in rabbits by upregulation of Bcl-2 and downregulation of the Bax protein in subarachnoid hemorrhage [68]. Therefore, investigation of the relationship between TMT-induction and H_2_ treatment and changes in Bcl-2 and Bax expression is intriguing [53,69]. Further, to explore the mechanism underlying the therapeutic effects of H_2_ inhalation in TMT-induced mice, we assessed the expression of Bcl-2 and the Bax protein. Our results showed that Bcl-2 expression was decreased in the TMT-only group, while Bax expression was increased in the TMT-only group. In contrast, H_2_ treatment reversed the alteration of Bcl-2 and Bax. Together, these data indicate that H_2_ inhalation ameliorated the TMT-induced toxicity and cognitive impairment in mice by inhibiting hippocampal apoptosis through Bcl-2 and Bax signaling pathways. Based on these overall findings, we can conclude that H_2_ administration in TMT-induced mice improved cognitive function and neurotoxic damage due to its antioxidative and anti-inflammatory effects.

## 4. Materials and Methods

### 4.1. Animals

Twenty-four male C57BL/6 mice (20–24 g, 8–9 weeks) were purchased from Orient Bio Inc. (Seongnam, Korea). The mice were kept in a pathogen-free environment (22 ± 2 °C with 50 ± 10% humidity) under a 12-h light/dark cycle. The mice were given rodent chow and filtered water ad libitum until the end of the experiment. The mice were acclimatized for 10 days in plastic cages (390 × 275 × 175 mm) with wood shaving bedding and were randomly divided into four different groups (n = 6 per group): non treated normal control group (NC), TMT-only injection group (TMT Only), TMT injection + lithium chloride (LC)-treated group as a positive control (PC), and TMT injection + 2% H_2_ inhalation-treated group (H_2_). All experimental procedures were performed in accordance with the protocol of the Institutional Animal Care and Use Committee, Yonsei University Wonju College of Medicine (Ethical approval no: YWC-191115-1).

### 4.2. Experimental Design

This experiment was designed as shown in Figure 1. TMT (Sigma–Aldrich, St. Louis, MO, USA) was dissolved in sterilized 0.9% saline, and a single dose of 2.6 mg/kg was injected ip into the mice except the NC group. For the PC group, lithium chloride (Sigma–Aldrich, St. Louis, MO, USA) (50 mg/kg body weight) was administered twice by ip injection at 0 and 24 h after TMT injection. Previous conducted studies have shown LC as a potent neuroprotective agent, found to ameliorate neurodegeneration, neuroinflammation, neurotoxicity, and behavioral disability in a TMT-induced hippocampal neurodegeneration model [70,71]. For the treatment of the H_2_ group, 2% H_2_ gas was produced from a H_2_-generating device designed and provided by the company (GOOTZ Co., Ltd., Suwon, Korea). The mice were administered 2% H_2_ gas for 30 min once a day for four weeks. To investigate the effect of H_2_ inhalation, seizure behaviors were observed for four consecutive days after TMT treatment. Furthermore, to assess the TMT-induced memory deficits, hippocampus-dependent memory tests (Y-maze memory test) were performed on the 7th day of TMT induction, at which time the seizure behavior had disappeared. Body weight was measured once per week for four weeks. At the end of the experiment, the mice were sacrificed using isoflurane anesthetic (Hana Pharm. Co., Hwaseong, Korea). After mice were sacrificed, their blood samples were collected from retro-orbital veins in EDTA tubes, and serum was separated by centrifugation at 14,000 rpm for 5 min at 4 °C. To obtain brain lysate samples, the heads of the mice were decapitated, and the hippocampal region of the brain was separated and homogenized in ice-cold RIPA lysis buffer (Pierce Biotechnology Inc., Rockford, IL, USA) with a protease inhibitor cocktail (Sigma Chemical Co., St. Louis, MO, USA) using the bead milling method (QIAGEN Tissue Lyser II, manufactured by Retsch., Goleta, CA, USA) at 30 frequency/sec for 10 min. Thereafter, the brain lysate homogenate was centrifuged at 14,000 rpm for 10 min at 4 °C, and the protein concentration of the obtained supernatant was checked using a bicinchoninic (BCA) protein assay kit (Takara Bio, Shiga, Japan) and normalized. Both collected serum and brain lysate samples were stored at −80 °C until further use. Furthermore, ROS, NO, Ca^2+^, MDA, GPx, and catalase activities were measured as OS-related markers. G-CSF, IL-1β, TNF-α, and VEGF were measured as inflammation-related markers, and Bcl-2 and Bax as apoptotic markers. In addition, Apo-E, Aβ-40, and p-tau were measured as AD-related markers.

### 4.3. Seizure Scoring and Body Weight Measurement

Seizure and tremor behavior tests were performed in mice until four days after TMT induction. Scores were assigned for behavioral changes in mice, ranging from 1 (aggression), 2 (weak tremor), 3 (systemic tremor), 4 (tremor and spasmodic gait), to 5 (death) [31]. In addition, body weight measurements were conducted every week for four weeks to obtain the baseline data of all experimental mice.

### 4.4. Y-Maze for Behavioral Test

Seven days after TMT injection, a Y-maze behavioral test was performed in mice according to their grouping. The maze was made from black Y-shaped plastic, and the arms were at an angle of 120° from each other. Each mouse was allowed to freely move in the maze for 8 min, and the sequence of arm entries was recorded with a Smart 3.0 video tracking system (Panlab, Cambridge, MA, USA). The spatial cognition (%) ability was calculated as follows: actual alternation / (total number of arm entries − 2) × 100. In addition, the total distance traveled in the zone was determined.

### 4.5. Detection of the ROS Level

ROS levels were measured using dichloro-dihydro-fluorescein diacetate (DCFH-DA) reagent (Sigma, St. Louis, MO, USA) following the manufacturer’s instructions. Briefly, 10 μL of serum and brain lysate samples and 100 μL of 10 µM DCFH-DA were mixed in a 96-well black plate and incubated for 30 min at 37 °C. The fluorescence was measured using a DTX multi-mode microplate reader (Beckman Coulter Inc., Brea, CA, USA) at 488 nm excitation/525 nm emission.

### 4.6. Detection of the NO Level

NO levels were measured using Griess reagent (iNtRON Biotechnology, Sungnam, South Korea) following the manufacturer’s instructions. Briefly, 50 μL of serum and brain lysate samples were mixed with an equal volume of Griess reagent in a 96-well microplate and incubated at room temperature for 15 min. The absorbance was measured at 540 nm using SpectraMax^®^ ABS Plus (Molecular Devices, San Jose, CA, USA). The NO_2_^−^ concentration was calculated by a standard curve graph that was generated by serial two-fold dilutions of sodium nitrate.

### 4.7. Analysis of Antioxidant Enzyme Activities

Intracellular levels of endogenous antioxidant enzymes (catalase and GPx) were measured using the BioVision kit (BioVision, Inc., Milpitas, CA, USA) following the manufacturer’s instructions. Briefly, 78 µL of samples (10 µL from stock and 68 µL assay buffer) for the catalase assay and 50 µL of samples (10 µL from stock and 40 µL assay buffer) for the GPx assay were added to a 96-well microplate, and the plates were incubated for 30 min. The optical densities of catalase (570 nm) and GPx (340 nm) were measured using SpectraMax^®^ ABS Plus (Molecular Devices, San Jose, CA, USA). The results of the catalase and GPx activities were expressed in nmol/min/mL and mU/mL.

### 4.8. Detection of Ca^2+^ Activity

Intracellular Ca^2+^ activities were measured in serum and brain lysates using a Ca^2+^ colorimetric assay kit (BioVision, CA, USA) following the manufacturer’s instructions. In brief, 50 µL of each sample was added to a 96-well microplate, and the plate was incubated for 30 min. The optical density was measured at 590 nm using SpectraMax^®^ ABS Plus (Molecular Devices, San Jose, CA, USA). The results were expressed in mM.

### 4.9. Detection of MDA Activity

The level of MDA in serum and brain lysates was measured using a thiobarbituric acid reactive substances assay kit (BioVision, Milpitas CA, USA). The assay was performed according to the manufacturer’s instructions. The reaction product was measured colorimetrically at 532 nm using the SpectraMax^®^ ABS Plus microplate reader (Molecular Devices, San Jose, CA, USA).

### 4.10. Detection of Inflammatory Cytokines and Vascular Endothelial Growth Factor by Multiplex Assay

Cytokine and chemokine profiling was performed using the Milliplex^®^ MAP Mouse Cytokine/Chemokine Magnetic Bead Panel 96-well plate assay (Millipore Corporation, Billerica, MA, USA) as a luminex-based multiplex technology. G-CSF, IL-1β, TNF-α, and VEGF were measured by using a multiplex immunoassay following the manufacturer’s protocol. In brief, each standard concentration was resuspended in standard diluents, and serial dilutions of the standard were prepared. The bead mixture was added to the standard and serum and brain lysates. The plate was incubated overnight (18 h) at 4 °C and was proceeded by a washing step. Detection antibody was added, and the plate was incubated at room temperature for 1 h. A streptavidin–phycoerythrin mix was added, and the plate was incubated at room temperature for 30 min. After the washing step, an assay buffer was added, and the plate was analyzed using the Luminex 200 Bio-Plex instrument.

### 4.11. Detection of Total Apo-E by ELISA

Apo-E levels in serum and brain lysates were detected using the Rat Apo-E ELISA kit (MyBioSource, Inc. San Diego, CA, USA), and analyses were performed according to the manufacturer’s instructions. The concentration of Apo-E was visualized at 450 nm using a SpectraMax^®^ ABS Plus spectrophotometer (Molecular Devices, San Jose, CA, USA), and the concentration of Apo-E was calculated using the standard curve.

### 4.12. Western Blot Analysis

The hippocampi of C57BL/6 mice were homogenized by using the bead milling method (QIAGEN Tissue Lyser II, manufactured by Retsch, Goleta, CA, USA) at 30 frequency/sec for 10 min, incubated with 500 µL lysis RIPA buffer at 4 °C, and centrifuged at 14,000 rpm for 10 min. The liquid supernatant was collected, and the BCA protein assay kit (Takara, Shiga, Japan) was used to measure the total protein concentration. Equal amounts of protein samples (20 µg) were separated on 12% polyacrylamide gels and transferred to a polyvinylidene difluoride membrane (Pall., Ann Arbor, MI, USA) at 300 mA for 2 h. After transferal, the membranes were blocked with a blocking buffer (Takara, Shiga, Japan) for 1 h at room temperature. The membranes were further incubated with the primary antibodies Beta-actin (Cell Signaling Technology, Danvers, MA, USA), Aβ-40 (My Biosource, San Diego, CA, USA), phospho-tau-(Ser404) (Cell Signaling Technology, Danvers, MA, USA), Bcl-2 and Bax (Cell Signaling Technology, Danvers, MA, USA) in a 1:2000 dilution at 4 °C overnight. After washing three times with 1× Tris Buffered Saline and Tween (1× TBST), the membranes were treated with horseradish peroxidase-(HRP) conjugated anti-rabbit secondary antibody (dilution 1:5000; Cell Signaling Technology) in 1× TBST for 2 h at room temperature. After washing three times, the bound antibodies were detected by an enhanced chemiluminescence kit (ECL Pierce Biotechnology, ThermoFisher Scientific, Waltham, MA, USA) using the UVP Bio Spectrum 600 Imaging System (UVP, LLC, Upland, CA, USA). Band intensity was analyzed using ImageJ software (Version 150-win Java, Bethesda, MD, USA).

### 4.13. Data Management and Statistical Analysis

Values are presented as the mean ± standard error of the mean (SEM). All data were analyzed and compared by two-way analysis of variance (ANOVA), followed by a multiple comparison test using the Graph Pad Prism 8.0 software package (Graph Pad, La Jolla, CA, USA). Differences were considered to be statistically significant at *p* < 0.05.

## 5. Conclusions

In summary, we evaluated the therapeutic effects of H_2_ gas inhalation on neurotoxicity and cognitive impairment in TMT-injected C57BL/6 mice. Our results showed that H_2_ gas inhalation effectively attenuated TMT-induced cognitive damage in mice. In addition, we found that H_2_ effectively decreased TMT-induced OS and inflammatory markers, such as ROS, NO, and MDA and inflammatory cytokines such as G-CSF, IL-1β, and TNF-α in the blood and brain lysates of TMT-induced mice. In addition, H_2_ increased VEGF levels in both the serum and brain in TMT-induced mice. Furthermore, H_2_ inhalation decreased AD signaling protein markers such as Apo-E, Aβ-40, and p-tau in the brain of TMT-induced mice. In addition, H_2_ ameliorated the TMT-induced toxicity in mice by regulating hippocampal apoptosis through Bcl-2 and Bax signaling pathways. However, further molecular mechanistic studies on different signaling pathways are required to investigate the detailed therapeutic role of H_2_ inhalation in TMT-induced neurotoxicity and cognitive damage. Taken together, our results strongly suggest that H_2_ treatment might potentially be a future therapeutic candidate for neurodegenerative diseases by improving the cognitive abilities and controlling OS and inflammation-mediated neurodegeneration.

## Data Availability

All of the data are contained within the article.
